# Supplementary material for: Learning curve and surgical outcome of robotic assisted colorectal surgery with ERAS program
Source: Sci Rep. 2022 Nov 29;12:20566. doi: 10.1038/s41598-022-24665-w (PMC9709162; doi:10.1038/s41598-022-24665-w)
Supplement: Supplementary file 1 — Supplementary Information 1. [file 41598_2022_24665_MOESM1_ESM.zip › LSC Raw data-Ñ[▒K/055/HS_ñw╝╨░O▒Kñσ.pdf]

正式摘要(電子簽章：000000182666964)

W95 / W95 ☐ 英文標題(注意:無英文姓名-請補病人基本檔!) ☐【讀取】憑證

臺中榮民總醫院

出院病歷摘要

轉入號： 轉出號：

[redacted]

(8)流水編號	
(9)入院日期	108/12/11 (2019) 直腸外科 W95 - 12
(10)轉科日期	
(11)出院日期	108/12/23 (2019) 住院天數計 12 日

診斷

- 入院:Rectal cancer 12cm from anal verge
- 出院:Rectal cancer, 12cm from anal verge, s/p Laparoscopic anterior resection on 2019/12/12, pT3N1b.

主訴

- Bloody stool noticed for weeks

病史

- This is a 39 years old male patient denied other underlying disease.
  According to the patient, he noticed that he had bloody stool in these weeks. He denied fever, abdominla pain, bowel habit change, dizziness, nausea or vomiting. Due to his sister had colon cancer history, he went to 埔榮 for further survey. Colonoscopy showed a rectal tumor, 12cm from anal verge. Labs showed CEA: 1.38.
  As mentioned above, he came to our OPD for second opinion. Operation was suggested and he agreed. Thus, he was admitted to our ward for surgery.

體檢發現

-
  - General appearance :
    - easy looking , conscious clear , without cardiopulmonary distress
    - Vital signs自動帶入date 體溫(BT) 脈搏(P) 呼吸(R) 血壓(BP) 身高(BH) 體重(BW) BMI
      20191211 36.7℃ 77次/分 18次/分 143/65mmHg 177cm 69kg 22.0
    - Vital signs: B.P. 143/65 mmHg , P.R. 77 /min , R.R. 18 /min , B.T. 36.7 ℃
  - HEENT :
    - Eyes: no anemic conjunctiva, no icteric sclera, normal EOM
    - ENT: no otorrhea, no rhinorrhea, no erythematous mucosa, no injected tonsil, no deformity
  - Neck: supple, freely movable, trachea midline,no deformity, no engorged jugular vein , no palpable thyroid or lymph node enlargement
  - Chest:
    - Inspection: Grossly normal, symmetric and fully expansion, no accessory muscle used, no operation scar, no spider angiomata, no gynecomastia
    - Palpation: no palpable mass or nodule, no subcutaneous emphysema, no tenderness
    - Ausculation: clear breathing sound
  - Heart:
    - Palpation: no thrill, no heave
    - Percussion: normal heart size
    - Ausculation: regular heart beat, normal S1, S2, no gallop, no murmur
  - Extremities: No deformity, full ranged of motion without limitation, no clubbing finger or nail cyanosis, no pitting edema, no ulcer or pigmentation, no obvious skin lesion, no varicose vein, no bone, joint or muscle tenderness

  CRS CONDITION

  - Karnofsky performance status: (100) Normal, no complaints, no evidence of disease

-
  - PE of Abdomen:
  - CEA: 1.38 ng/ml
  - Imaging Studies:
  - Colonoscope report: RECTAL CA NOTED OVER 12CM FAV
  - Figures:
    1. colon & rectum

手術日期及方法

- 手術日期及方法20191212 Low anterior resection +/- pelvic LN dissection Laparoscopy
- 手術結果及發現 :

住院治療經過

- <12/11~12/17> After admission, the patient received Lapraoscopic anterior resection on 2019/12/12. His SBP range was about 88~91 at POR and Penrose drain showed dark red fluid, but Hb was 10.2, so he was sent to ordinary ward on 12/12 night. On 12/13, FFP was given for fluid loss during operation, but allergy reaction appears, so we stop FFP and gave steroid. Post OP day 2, he had flatus so start to try water. In addition, he received penrose revision due to suspect wound bleeding on 12/14. Hb showed 8.0 on 12/15, so we arrange blood transfusion pRBC 2U. PPI pump and Transamin and Vit. K1 were used. Diarrhea on 12/16 night. <12/18~12/23> During this week, he had penrose drain revision 2cm 3 times. We keep follow up clinical symptoms and labs data. Due to stable condition and vital sign, Hb keep at 10.2, thus, we let him discharge on 12/23 and arrange OPD follow up.

合併症

一般檢查

- 累積報告 :

DATE	TIME	ALB	TP	BIL.T	BIL.D	ALKP	AST	ALT	LDH	CHOL	TG	HDL-C	LDL	UA	NH3	AMY
1081212	1823	4.0		0.7	0.2		13	12								

DATE	TIME	WBC	RBC	HGB	HCT	MCV	PLT	BLST	NEUT	BAND	SEG	LYM	MON	EOS	BAS
1081211	2151						282								
1081212	1821	12020	4.12	13.2	40.0		256		85.3			7.5	6.6	0.4	0.2
1081212	2317			10.7											
1081213	0917	9900	3.08	10.2	30.0	97.4	233		70.9			13.1	15.7	0.2	0.1
1081215	0952	5170	2.51	8.0	24.8	98.8									
1081216	0955	6990	3.03	9.6	29.3	96.7	246		65.4			21.6	8.3	4.4	0.3
1081219	0940	7670	3.16	10.2	30.2	95.6	267		73.1			11.9	9.5	5.2	0.3
1081222	1010	7610	3.23	10.2	31.0	96.0	360		66.0			14.8	10.0	8.9	0.3

DATE	TIME	NA	K	CL	CA	BUN	CREAT	Mg	P	CRP	ESR	PCT	LIPASE	LACTATE
1081211	1914	139	4.1			9	0.81							
1081212	1823	140	4.1			8	0.89							
1081213	0925	140	3.9			7	0.92							
1081216	0855	139	4.5			11	0.66							
1081222	1014	138	4.7			9	0.79							

DATE	TIME	GLUCOSE	PAB	TRF	ZN	CU	KETONE	CK	CKMB	PT-P	PT-C	APTT-P	APTT-C	TRO-T
1081219	0932									11.7	10.1	28.8		

- 放射線報告 :**檢驗項目 :CHEST PA VIEW** 報告日期 **:1081215**

  主　訴：

  Result:

  Finding:

  1. No cardiomegaly.
  2. Mild scoliosis and spondylosis of thoracolumbar spine.
  3. No active lung lesion.
  4. Otherwise, no other significant finding.

  Impression:

  As above description.

特殊檢查

病理報告

- 活體組織切片病理報告　手術日期：**108/12/13**　病理號：**S0844662**

診斷醫師:吳致瑩 報告日期:108/12/19

- Pathologic diagnosis:
  1. Rectum, laparoscopic low anterior resection --- Adenocarcinoma, moderately differentiated. Tumor invaded to perirectal soft tissue, and metastasized to perirectal lymph nodes (2/17). The proximal cut end is free of tumor.
  2. IMA root, dissection --- Unremarkable soft tissue and lymph nodes (0/8).
  3. Rectum, cut end, distal, resection --- Unremarkable.
  4. Lymph node, IMA, dissection --- Unremarkable lymph nodes (0/3).
  Ancillary study for diagnosis:
  IHC stains for MLH1, MSH2, PMS2 and MSH6 are done on section E. All above markers are preserved.
  Prognostic and predictive factor:
  1. Tumor size: 3.8x2 cm.
  2. Depth of invasion: Perirectal soft tissue.
  3. Histologic grade: G2: Moderately differentiated.
  4. Angiolymphatic invasion: Not identified.
  5. Perineural invasion: Present.
  6. Tumor deposits (discontinuous extramural extension): Not identified.
  7. Surgical Margins: a) Proximal Margin: Uninvolved by invasive carcinoma, distance of tumor from margin: 5.5 cm. b) Distal Margin: Uninvolved by invasive carcinoma, distance of tumor from margin: 2 cm. c) Radial or Mesenteric Margin: Uninvolved by invasive carcinoma, distance of tumor from margin: 1.7 cm.
  8. Lymph node status: Involved (2/28).
  9. Extranodal involvement: Present.
  10. Tumor regression grading S/P CCRT: No known presurgical therapy.
  11. Type of polyp in which invasive carcinoma arose: Not applicable.
  12. Additional pathologic findings: Not identified.
  13. Pathological TNM Stage: pT3N1b (According to the eighth edition, 2017, American Joint Committee on Cancer Staging Guidelines for Tumors / 2017 CAP guideline).
  14. TNM descriptors: Not applicable.
  Gross description:
  The specimen consists of 1) a segment of rectum, 13 cm in length and 4 cm in proximal cut end circumference, 6.5 cm in distal circumference, and perirectal soft tissue. A gray tan firm annular elevated and centrally ulcerative tumor, 3.8x2 cm, is noted, 2 cm to the distal cut end. On cross section, the tumor invades to the mesocolic soft tissue with 17 mm to the radial margin, and serosal retraction is also noted. The both cut ends are free of tumor grossly. 2) IMA root, a piece of tan yellow soft tissue, 4x1.4x0.4 cm. 3) distal cut end, a piece of tan mucosa-covered soft tissue, 2x1.5x1 cm. 4) a piece of tan gray soft tissue, 0.3x0.3x0.2 cm, labeled as "IMA LN". All for section: A) specimen 2 C) specimen 4; Representative part for section: B) specimen 3 D) proximal cut end E-G) tumor with radial cut end and serosa H-K) LN.
  Note:
  1. The interpretation of MMR panel and recommended studies are as below: (1) No loss of nuclear expression of MMR proteins: low probability of microsatellite instability-high (MSI-H) . (2) Loss of nuclear expression of MLH1 and PMS2: testing for methylation of the MLH1 promoter and/or mutation of BRAF is indicated (the presence of a BRAF V600E mutation and/or MLH1 methylation suggests that the tumor is sporadic and germline evaluation is probably not indicated; absence of both MLH1 methylation and of BRAF V600E mutation suggests the possibility of Lynch syndrome, and sequencing and/or large deletion/duplication testing of germline MLH1 may be indicated). (3) Loss of nuclear expression of MSH2 and MSH6: high probability of Lynch syndrome (sequencing and/or large deletion/duplication testing of germline MSH2 may be indicated, and, if negative, sequencing and/or large deletion/duplication testing of germline MSH6 may be indicated). (4) Loss of nuclear expression of MSH6 only: high probability of Lynch syndrome (sequencing and/or large deletion/duplication testing of germline MSH6 may be indicated). (5) Loss of nuclear expression of PMS2 only: high probability of Lynch syndrome (sequencing and/or large deletion/duplication testing of germline PMS2 may be indicated). (6) Please note: There are exceptions to the above IHC interpretations. These results should not be considered in isolation, and clinical correlation with genetic

- counseling is recommended to assess the need for germline testing.
  2. This case has been peer reviewed by two doctors.
  #T-59600_2 #M-81403_2 2 1105 S0000F
  #T-C4520_2 #M-81406_2 2 1105 000000

其它

出院時情況

- 3.改門診治療

出院指示

-

藥名	劑量	劑量單位	用法	頻次	數量	數量單位	現況	天數	單位
Ulstop tab 20mg	1	TAB	PO	BID	14	TAB	TH	7	天
Ultracet tab	1	TAB	PO	Q8HPRN	21	TAB	TH	7	天

出院計劃

- 門診日期
  - date on 2020/01/02
- 衛教 :Post OP diet Medication use
- Arrange OPD follow up May need chemoport insertion next time

主治醫師 2209J 陳志典 / 住院醫師 2339C 蔡濬樟

正式摘要(電子簽章：000000182666964)

W95 / W95

doctorRank:B
